# Identification and characterization of two AA9 lytic polysaccharide monooxygenases isolated from the enzymatic arsenal in *Phanerochaete* *chrysosporium*

**DOI:** 10.1186/s40643-025-00950-0

**Published:** 2025-10-10

**Authors:** Xiaofeng Xu, Chong-En Chu, Nuo Li, Xiao-Bao Sun, Junyan Han, Zihan Xu, Yu Liu, Jia-Kun Wang, Tie-Tao Zhang, Qian Wang

**Affiliations:** 1 Key Laboratory of Molecular Animal Nutrition, Ministry of Education, Zhejiang University, Hangzhou, 310058 China; 2 Institute of Dairy Science. College of Animal Sciences, Zhejiang University, Hangzhou, 310058 China; 3 College of Biological and Environmental Sciences, Zhejiang Wanli University, Ningbo, 315100 China; 4 College of Life Sciences, Zhejiang University, Hangzhou, 310058 China; 5 Specialty Research Institute, Chinese Academy of Agricultural Sciences, Changchun, 130112 China

**Keywords:** *Phanerochaete chrysosporium*, LPMO, Lignocellulose deconstruction, Synergistic effect

## Abstract

White rot fungi including *Phanerochaete chrysosporium* are known for their ability to mineralize plant-derived materials, such as cellulose, hemicellulose, and lignin, into CO_2_ and H_2_O. This process is achieved through a diverse array of hydrolytic and oxidative enzymes. However, the mode of action and specific characteristics of lytic polysaccharide monooxygenases (LPMOs) from *P. chrysosporium* are not well understood. In this study, two auxiliary activity (AA) family 9 genes from *P. chrysosporium*, *PchAA9C* and *PchAA9F*, were heterologously expressed in *Pichia pastoris* and functionally characterized. The recombinant PchAA9C and PchAA9F exhibited optimal activity at 60 °C and pH 6.0, with their activity significantly enhanced by 0.5–3.0 mmol/L ascorbic acid (*P* < 0.05). Substrate specificity analysis revealed that both PchAA9C and PchAA9F displayed robust activity against Icelandic moss lichenan, phosphoric acid swollen cellulose, and microcrystalline cellulose, indicating a preference for breaking down β-(Glc1 → 4Glc)-linked substrates. Further analysis using HPAEC-PAD and MALDI-TOF-MS revealed that PchAA9C functioned as a C1-specific oxidizing enzyme, whereas PchAA9F targeted both C1 and C4 positions of sugar rings. Synergistic experiments involving an enzyme cocktail of xylanase, glucanase, and pectinase showed that PchAA9C and PchAA9F significantly enhanced the production of reducing sugars from corn and soybean straws. Notably, PchAA9F represents the first reported C1/C4-double-oxidizing LPMO isolated from *P. chrysosporium*. This discovery provides new insights into the molecular basis of the biodegradation capabilities of wood-decaying fungi and highlights PchAA9F as a promising candidate for applications in lignocellulosic biomass biorefinery.

**Graphical abstract:**

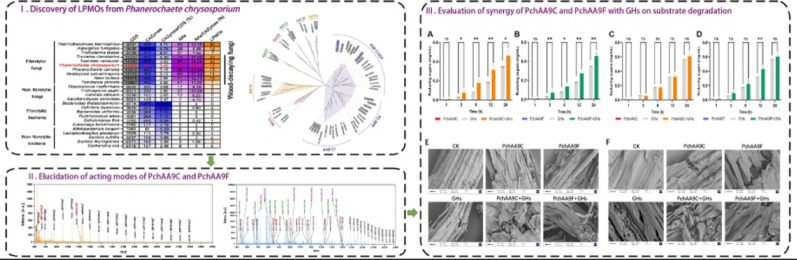

**Supplementary Information:**

The online version contains supplementary material available at 10.1186/s40643-025-00950-0.

## Introduction

Plant-derived lignocelluloses, the most abundant renewable biomass in nature, have gained significant interest for producing value-added commodities such as biofuels, functional foods, and feed additives (Vaaje-Kolstad et al. 2010; Uchiyama et al. 2022). However, plant cell wall, referred to as biomass barrier, is highly resistant to deconstruction. Microbial degradation of plant-derived biomass typically involves free enzymes secreted extracellularly or specialized surface-associated systems like cellulosome and polysaccharide utilization loci. The enzymes responsible for lignocellulose degradation primarily include glycoside hydrolases (GHs), polysaccharide lyases (PLs), carbohydrate esterases (CEs), and non-catalytic carbohydrate-binding modules (CBMs). Among them, GHs play pivotal roles in breaking down plant polysaccharides and are widely applied in the food, feed, and biorefinery industries (Rojas-Pérez et al. 2022; Elshami et al. 2025).

Lytic polysaccharide monooxygenases (LPMOs, EC 1.14.99.53–56), formerly classified as GH61 and CBM33 based on negligible hydrolytic activity, represent a unique class of enzymes that degrade substrates via an oxidative mechanism, mainly including C1-, C4-, and C1/C4-acting modes (Singhania et al. 2021). In late 2011, these enzymes were reclassified from GH61 and CBM33 to polysaccharide monooxygenases (PMOs) and subsequently as LPMOs, forming part of the auxiliary activities (AAs) subfamilies. According to the Carbohydrate-Active Enzymes (CAZy) database (https://www.cazy.org/), LPMOs are categorized into families AA9-11 and AA13-17 (Drula et al. 2022). Structurally, LPMOs exhibit an immunoglobulin- or fibronectin type III-like β-sandwich fold. Unlike traditional GHs, their active sites are located on a flat surface of β-sheet rather than within substrate-binding cavities or tunnels (Couturier et al. 2018).

The emergence of LPMOs has enhanced polysaccharide deconstruction by introducing oxidative chain breaks, enabling GHs to further degrade oligosaccharides. Notably, certain LPMOs, such as PchAA9E and LsAA9A, can also capable of degrading oligosaccharides (Frandsen et al. 2021). Despite variations in their origins and modes of action, studies show that LPMOs effectively boost the saccharification efficiency of GHs and hold potential for consolidated bioprocessing of plant-derived biomass. Their robust oxidative capabilities towards diverse lignocellulosic substrates have driven efforts to identify novel LPMOs with high activity and desired properties. Over the past decade, a series of LPMOs have been shown to synergize with other carbohydrate-active enzymes (CAZymes), such as GHs and PLs, to degrade various polysaccharides, including cellulose (Xin et al. 2022), chitin (Nakagawa et al. 2020; Zhao et al. 2024), glucan (Agger et al. 2014; Simmons et al. 2017; Sun et al. 2023), pectin (Sabbadin et al. 2021), and xylan (Simmons et al. 2017; Corrêa et al. 2019; Tõlgo et al. 2023) from bacteria, fungi, virus, archaea, and insects.

Basidiomycetes, the second most abundant phylum after Ascomycetes, comprise more than 40,000 species (Konan et al. 2024). These fungi include key wood-decaying species such as white rot fungi (e.g. *Phanerochaete chrysosporium*, *Trametes versicolor*, *Phanerochaete carnosa*, *Gelatoporia subvermispora*, and *Irpex lacteus*) and brown rot fungi (e.g. *Trametes cinnabarina*, *Fomitopsis pinicola*, and *Laetiporus sulphureus*). Their genomes are remarkable resources for understanding enzymatic lignocellulose breakdown mechanisms (Suzuki et al. 2012; Qin et al. 2022). Among these, *P. chrysosporium* is a model microorganism for white rot fungi, known for its ability to degrade all lignocellulosic materials. It is also extensively used for degrading substances like thiamethoxam (Zhu et al. 2024), melanin (Sadaqat et al. 2020), atrazine, and fipronil (Kumari et al. 2023). These capabilities are largely attributed to its production of CAZymes, particularly lignolytic enzymes (Konan et al. 2024) such as laccases, manganese peroxidases, and lignin peroxidases, which are exclusively distributed within the AA1, AA2 and AA2 families (Levasseur et al. 2013), respectively. Due to its effectiveness in biomass bioconversion, *P. chrysosporium* has attracted significant interest for discovering novel lignocellulose-degrading enzyme consortia. Over the years, multi-omics strategies, such as transcriptome and secretome (Ravalason et al. 2008; Wymelenberg et al. 2009; Mahajan and Master. 2010; Machado et al. 2020) have been employed to unravel the molecular and enzymatic mechanisms of lignocellulose degradation. However, the biochemical characteristics of *P. chrysosporium* remain incompletely understood. To further explore the activity of white-rot-fungi-derived LPMOs, two novel AA9 LPMOs, PchAA9C and PchAA9F, from *P. chrysosporium* were chosen and functionally characterized.

## Materials and methods

### Materials

Microbial strains *Escherichia coli* DH5α, *E. coli* BL21(DE3), *Pichia pastoris* GS115 (*his4*), and expression vectors including pET-28a(+), pET-30a(+) and pPIC9K were stored in our laboratory. The *P. chrysosporium* CICC40299, was obtained from the China Center of Industrial Culture Collection. Culture media, kanamycin, isopropyl-thio-β-D-galactopyranoside (IPTG), and Ni-TED Sepharose 6FF (His-Tag) were obtained from Sangon (Shanghai, China). Icelandic moss lichenan (IML), and cello-oligosaccharides were purchased from Megazyme (Wicklow, Ireland). Phosphoric acid swollen cellulose (PASC) was prepared from microcrystalline cellulose (MCC) as previously described (Zhang et al. 2006). *Zea mays* L. and *Glycine max* L. straws were collected from local farms in Zhejiang, China.

### Comparative genome analysis of P. chrysosporium and phylogeny analysis of PchAAs

Genomic data were retrieved from the NCBI datasets portal (https://www.ncbi.nlm.nih.gov/datasets/genome/). This study provides comprehensive analysis of genome information from 25 microorganisms: fibrolytic fungi (10 species: *Thermothelomyces thermophilus*, *Aspergillus fumigatus*, *Trichoderma reese*i, *T. cinnabarina*, *T. versicolor*, *P. chrysosporium*, *P. carnosa*, *G. subvermispora*, *I. lacteus*, and *F. pinicola*), non-fibrolytic fungi (four species: *Cryptococcus neoformans*, *Trichosporon asahii*, *Candida albicans*, and *Saccharomyces cerevisiae*), fibrolytic bacteria (six species: *Bacteroides thetaiotaomicron*, *Cellvibrio japonicus*, *Bacteroides uniformis*, *Ruminococcus albus*, *Cellulomonas fimi*, and *Cytophaga hutchinsonii*), and non-fibrolytic bacteria (five species: *Bifidobacterium longum*, *Lactiplantibacillus plantarum*, *Bacillus subtilis*, *Bacillus thuringiensis* and *E. coli*). The analysis included genome size, GenBank assembly accession, coding sequences (CDS), CAZymes, AAs family, and LPMOs. Data were sourced from genome and enzyme databases, including CAZy (https://www.cazy.org/Home.html) and DOE-JGI (Joint Genome Institute).

Multiple sequence alignment of LPMOs was performed using MUSCLE in MEGA software (v.6.0, Kumer Lab, Temple University, Philadelphia, USA). Phylogeny analysis of PchAAs were conducted using IQ-Tree v2.2.0 with a maximum-likelihood approach (1000 rapid bootstrap replicates) under the PMB + F + R4 model (Minh et al. 2020). The resulting phylogenetic tree was visualized using iTol (https://itol.embl.de/) (Letunic and Bork. 2021). Structural models of PchAA9C and PchAA9F were generated using SWISS-MODEL (Waterhouse et al. 2018), based on the crystal structures of *Mc*AA9F from *Malbranchea cinnamomea* (PDB: 7NLT) and *Hi*LPMO9B from *Heterobasidion irregulare* (PDB: 5NNS), respectively. The cellohexaose-bound complex was modeled by superimposing the structure with the LsAA9A-cellohexaose complex from *Lentinus similis* (PDB: 5ACI). The structural images were prepared using PyMOL (Version 4.6).

### Cultivation and RNA isolation of P. chrysosporium

*P. chrysosporium* spores were inoculated onto 2% (w/v) malt extract (1%) agar slants and incubated at 24 °C for 3–4 weeks (Kirk et al 1978). Spores were scraped with a sterile spatula and lysed in sterile water. Approximately 5.0 × 10^6^ spores were inoculated into Vogel’s medium (0.25% Na_3_C_6_H_5_O_7_·2H_2_O, 0.5% KH_2_PO_4_, 0.2% NH_4_NO_3_, 0.02% MgSO_4_, 0.01% CaCl_2_, 0.01% trace elements solution, and 0.1 μg/mL biotin, pH 4.5) with 1% corn straw as the sole carbon source. Cultures were incubated at 39 °C and 125 rpm for 5 d to induce fibrolytic enzyme gene expression. Mycelia were harvested by centrifugation at 5,500 rpm for 10 min, and total RNA was extracted using a fungal total RNA isolation kit (Sangon Biotech, Shanghai, China)*.* Approximately 1 μg of total RNA was used for reverse transcription with the HiScript® Q RT SuperMix kit (Vazyme, Nanjing, China).

### Gene cloning, expression and protein purification

Two candidate genes, *PchAA9C* and *PchAA9F,* were amplified from *P. chrysosporium* cDNA using gene-specific primers (Table S1) and Phanta SE Super-Fidelity DNA polymerase (Vazyme, Nanjing, China). The purified DNA fragments were inserted into the shuttle vector pPIC9K using the 2 × MultiF Seamless Assembly Mix (Vazyme, Wuhan, China). The resulting plasmids were transformed into *E. coli* DH5α by heat shock and selected on LB agar plates (0.5% yeast extract, 1% peptone, 1% NaCl, and 2% agar) containing 50 μg/mL kanamycin. For *P. pastoris* integration, approximately 10 μg of *Bgl*II-linearized recombinant plasmids were electroporated (1500 V, 4.6 ms, Eppendorf 2510, USA) into GS115 cells. Transformants were cultured on MD plates (1.34% yeast nitrogen base, 0.4 μg/mL biotin, 2% dextrose, and 2% agar) and MM plates (1.34% yeast nitrogen base, 0.4 μg/mL biotin, 0.5% methanol, and 2% agar) at 30 °C for 2–3 d. Positive transformants were confirmed by PCR amplification using α-factor and 3’AOX primers. Recombinant yeasts, GS115/pPIC9K-*PchAA9C*, and GS115/pPIC9K-*PchAA9F*, were cultured in BMGY medium (1% yeast extract, 2% peptone, 1.34% yeast nitrogen base, 0.4 μg/mL biotin, 1% glycerol, and 0.1 mol/L potassium phosphate, pH 6.0) and induced in BMMY medium (1% yeast extract, 2% peptone, 1.34% yeast nitrogen base, 0.4 μg/mL biotin, 1% methanol, and 0.1 mol/L potassium phosphate, pH 6.0) for protein expression (Wang et al 2011).

Yeast cells cultured for 96 h were harvested by centrifugation at 8000 rpm for 15 min. The culture supernatants were collected and used for 6 × His-tagged protein purification using a linear imidazole gradient (20–1000 mmol/L) on an ÄKTA start protein purification system (GE Healthcare Life Sciences, Pittsburgh, PA, USA) as described previously (Li et al 2024). Purified proteins were further filtered through a 10-kDa regenerated cellulose membrane (Millipore, Billerica, MA, USA) to remove imidazole. For deglycosylation, ~ 20 μg of purified PchAA9C or PchAA9F was treated with 500 U PNGase F (Vazyme, Nanjing, China) and incubated at 37 °C for 24 h.

Three *E. coli* BL21(DE3) harboring pET-28a( +)-Cel5A-h38 (Cao et al. 2021), pET-30a( +)-XYN-LXY (Wang et al. 2015) and pET-30a(+)-IDSPGA28-16 (Deng et al. 2023) were used for the preparation of recombinant glucanase, xylanase and polygalacturonase. The *E. coli* BL21(DE3) cells were induced with IPTG, harvested, sonicated and purified as described in our previous study (Li et al. 2024). Both native and deglycosylated proteins were analyzed by sodium dodecyl sulfate–polyacrylamide gel electrophoresis (SDS-PAGE; 15% running gel and 4% stacking gel) and Western blotting as described previously (Wang et al. 2012). Protein bands were visualized with a Feto SDS-PAGE staining buffer (Aidisheng, Yancheng, China).

### Activity assays

Protein concentrations were determined using the Bradford method (Bradford 1976). Prior to activity assays, recombinant PchAA9C and PchAA9F were pretreated for copper saturation by preincubating each enzyme with CuSO_4_ (molar ratio = 1:3) for 60 min on ice. Unbound copper ions were removed by ultrafiltration using an Amicon ultra centrifugal filter, followed by resuspension of the copper-saturated enzymes in 50 mmol/L sodium acetate buffer (pH 5.5). To assess the copper-binding status of PchAA9C and PchAA9F, copper content was quantified using inductively coupled plasma mass spectrometry (ICP-MS) on a NexION 300D system (PerkinElmer, Shelton, USA) operated at m/z = 63 (Skalnyet al. 2020).

Activity assays were estimated using the 3,5-dinitrosalicylic acid (DNS) method (Sun et al. 2023). 15 μL of copper-saturated enzyme (~ 0.6 μg) was mixed with 60 μL of 5 mg/mL polysaccharide substrates (IML, PASC, and MCC) dissolved in 50 mmol/L sodium phosphate buffer (pH 5.5) and incubated at 37 °C with 200 rpm for 24 h. No ascorbic acid or H₂O₂ was added during the reaction. Following incubation, 75 μL of DNS was added, and the mixture was heated at 99 °C for 10 min. After cooling to room temperature, absorbance was measured spectrophotometrically at 540 nm. One unit (U) of LPMO activity was defined as the amount of enzyme required to release 1 μmol of reducing sugar per hour. All experiments were carried out in triplicate unless otherwise specified.

### Biochemical characterization of PchAA9C and PchAA9F

To determine the optimal pH and temperature profiles of PchAA9C and PchAA9F, assays were conducted by incubating 15 μL of each enzyme (~ 0.6 μg) with 60 μL of 5 mg/mL IML substrate dissolved in diverse buffers of pH 2.2–8.0 (pH 2.2–3.0, glycine–HCl; pH 4.0–6.0, sodium acetate; pH 7.0–8.0, phosphate) (Di Domenico et al. 2025). Reactions were carried out at temperatures ranging from 30 to 70 °C for 24 h in the absence of ascorbic acid or H₂O₂. Total reducing sugar release was quantified using the DNS method as previously described. The treatment yielding the highest activity was established as 100%.

To investigate the effects of ascorbic acid and H_2_O_2_ on PchAA9C and PchAA9F, the enzymes were incubated at 37 °C for 24 h in 5 mg/mL IML containing different concentrations of ascorbic acid (0.5, 1, 1.5, and 3 mmol/L) and H_2_O_2_ (0.01, 0.02, 0.05, 0.1, 0.2, 0.5, 1, 2.5, 5, and 10 mmol/L). Relative enzyme activities were estimated.

### Catalytic products of PchAA9C and PchAA9F

To explore the degradation products of PchAA9C and PchAA9F, reactions were conducted using 5 mg/mL IML as the substrate, 1.5 μmol/L PchAA9C and PchAA9F (~ 38 μg), and 1.5 mmol/L ascorbic acid at 30 °C for 48 h. The reactions were inactivated by boiling for 5 min, followed by centrifugation at 12,000 rpm for 15 min at 4 °C. The supernatants were analyzed using high performance anion-exchange chromatography with a pulsed amperometric detector (HPAEC-PAD, ICS3000, Dionex) equipped with a CarboPac PA200 analytical column (3 × 250 mm) and a guard column (3 × 50 mm) Thermo), and MALDI-TOF-MS (UltrafleXtreme, Bruker, Germany), following established protocols (Sun et al. 2023; Westereng et al. 2013).

### Synergy of PchAAs and GHs on the degradation of natural lignocellulosic feedstocks

Prior to enzymatic deconstruction, natural lignocellulosic feedstocks, including *Z. mays* L. and *G. max* L. straws, were pretreated with 2% NaOH as described by Cao et al. (2021). A fibrolytic enzyme cocktail was prepared by combining a variety of GHs derived from rumen microbiota previously obtained by our group, including a bifunctional endo-/exo-xylanase XYN-LXY (Wang et al. 2015), a bifunctional endo-/exo-glucanase Cel5A-h38, and an exo-polygalacturonase IDSPGA28-16 (Deng et al. 2023).

To investigate the synergistic effects of PchAA9C or PchAA9F and the GH cocktail (XYN-LXY:Cel5A-h38:IDSPGA28-16 = 1:1:1) on the degradation of *Z. mays* L. and *G. max* L. straws, 1.0 mmol/L ascorbic acid, equivalent amount (2 μmol/L for each enzyme) of PchAA9C or PchAA9F combined with GH cocktail (LPMO:XYN-LXY:Cel5A-h38:IDSPGA28-16 = 1:1:1:1) were added to 0.05 g of pretreated *Z. mays* L. and *G. max* L. straws in 2 mL phosphate-buffered saline (PBS, pH 7.4) at 30 °C for 24 h. Aliquots of the supernatant were collected at 1, 3, 6, 12, and 24 h, and the reducing sugars were quantified using the DNS method. Insoluble materials were rinsed ten times with ddH_2_O, dried, and used for scanning electron microscopy (SEM) analysis (Deng et al 2024).

## Results and discussion

### P. chrysosporium employs a series of LPMOs to depolymerize lignocellulosic substrates

To gain an overall understanding of the molecular basis of fungi and bacteria, we conducted a comparative genome analysis of fibrolytic fungi, non-fibrolytic fungi, fibrolytic bacteria, and non-fibrolytic bacteria (Table 1). As expected, the ten fibrolytic fungi included in this study contained significantly greater total numbers of genomic CAZymes (384–555 vs. 149–237) and higher ratios of CAZymes/CDS (2.92–5.76 vs. 2.47–3.09) compared with non-fibrolytic fungi. A similar phenomenon was observed between fibrolytic and non-fibrolytic bacteria. Strikingly, *B. thetaiotaomicron*, a model gastrointestinal tract species inhabiting the intestines of humans and animals, displayed the highest CAZymes/CDS ratio of 10.09% among the 25 species evaluated in this study, which is largely attributed to its relatively smaller genome size compared with eukaryotic cells (Table 1). The *B. thetaiotaomicron*, *B. uniformi* and *R. albus* are known gut microbes that stably colonize the gastrointestinal tracts of human and animals. The absence of AAs in their genome of these gut microbes is likely a result of long-time adaption to the anaerobic conditions of the gut environment. On the other hand, some non-fibrolytic microbes encode a limited number of LPMOs, such as CnCel in *Cryptococcus neoformans* (Probst et al. 2023) and CbpD in *Pseudomonas aeruginosa* (Askarian et al.^2021^), which are thought to contribute to pathogenicity rather than lignocellulose degradation, likely by remodeling their own cell walls during host infection.

**Table 1:** Genome information of fibrolytic and non-fibrolytic microorganisms deposited in the CAZy database

Classification	Species	NCBI accession	Genome size (Mb)	CDS^a^	CAZymes^>b^	CAZymes/CDS(%)	AAs^c^	AAs/ CAZymes (%)	LPMOs^d^								
									AA 9	AA 10	AA 11	AA 13	AA 14	AA 15	AA 16	AA 17	Total
Fibrolytic fungi	*Thermothelomyces thermophilus* ATCC 42464	GCA_000226095.1	38.7	9097	428	4.70	53	12.38	22	0	4	1	0	0	3	0	30
	*Aspergillus fumigatus* Af293	GCA_000002655.1	29.4	9630	555	5.76	38	6.85	7	0	3	0	1	0	1	0	12
	*Trichoderma reese*i QM6a	GCA_000167675.2	33.4	9109	411	4.51	31	7.54	3	0	3	0	1	0	0	0	7
	*Trametes cinnabarina* BRFM137	GCA_000765035.1	33.7	10,233	440	4.30	75	17.05	17	0	0	0	4	0	0	0	21
	*Trametes versicolor* FP-101664 SS1	GCA_000271585.1	44.8	14,292	503	3.52	94	18.69	18	0	0	0	4	0	0	0	22
	*Phanerochaete chrysosporium* RP-78	GCA_000167175.1	35.2	13,602	462	3.40	93	20.13	16	0	0	0	2	0	0	0	18
	*Phanerochaete carnosa* HHB-10118-sp	GCA_000300595.1	46.3	13,918	443	3.18	95	21.44	11	0	0	0	2	0	0	0	13
	*Gelatoporia subvermispora* B	GCA_000320605.2	39.0	12,122	384	3.17	66	17.19	9	0	0	0	2	0	0	0	11
	*Irpex lacteus* CCBAS Fr 238 617/93	GCA_022606095.1	47.7	15,316	452	2.95	71	15.71	16	0	0	0	3	0	0	0	19
	*Fomitopsis pinicola* FP-58527 SS1	GCA_000344655.2	41.6	13,851	405	2.92	47	11.60	4	0	0	0	3	0	0	0	7
Non-fibrolytic fungi	*Cryptococcus neoformans* var. neoformans JEC21	GCA_000091045.1	19.1	6632	205	3.09	17	8.29	1	0	1	0	1	0	0	0	3
	*Trichosporon asahii* var. asahii CBS 2479	GCA_000293215.1	24.5	8311	237	2.85	27	11.39	0	0	1	0	4	0	0	0	5
	*Candida albicans* SC5314	GCA_000182965.3	14.3	6030	160	2.65	10	6.25	0	0	0	0	0	0	0	0	0
	*Saccharomyces cerevisiae* S288C	GCA_000146045.2	12.1	6021	149	2.47	7	4.70	0	0	0	0	0	0	0	0	0
Fibrolytic bacteria	*Bacteroides thetaiotaomicron* DSM 2079	GCA_014131755.1	6.3	4718	476	10.09	0	0.00	0	0	0	0	0	0	0	0	0
	*Cellvibrio japonicus* Ueda107	GCA_000019225.1	4.6	3620	331	9.14	2	0.60	0	2	0	0	0	0	0	0	2
	*Bacteroides uniformis* CL03T12C37	GCA_018292165.1	4.9	3936	304	7.72	0	0.00	0	0	0	0	0	0	0	0	0
	*Ruminococcus albus* DSM 20455	GCA_000179635.2	4.5	4001	284	7.10	0	0.00	0	0	0	0	0	0	0	0	0
	*Cellulomonas fimi* ATCC 484	GCA_000212695.1	4.3	3783	240	6.34	1	0.42	0	1	0	0	0	0	0	0	1
	*Cytophaga hutchinsonii* ATCC 33406	GCA_000014145.1	4.4	3700	177	4.78	0	0.00	0	0	0	0	0	0	0	0	0
Non-fibrolytic bacteria	*Bifidobacterium longum* subsp. longum JCM 1217	GCA_000196555.1	2.4	1903	97	5.10	0	0.00	0	0	0	0	0	0	0	0	0
	*Lactiplantibacillus plantarum* SRCM100442	GCA_009913655.1	3.2	2929	111	3.79	1	0.90	0	1	0	0	0	0	0	0	1
	*Bacillus subtilis* subsp. subtilis str. 168	GCA_000009045.1	4.2	4237	156	3.68	0	0.00	0	0	0	0	0	0	0	0	0
	*Bacillus thuringiensis* ATCC 10792	GCA_000161615.1	6.3	6171	160	2.59	3	1.88	0	3	0	0	0	0	0	0	3
	*Escherichia coli* O157:H7 str. Sakai	GCA_000008865.2	5.6	5115	127	2.48	0	0.00	0	0	0	0	0	0	0	0	0

^a^CDS, coding sequence; ^b^CAZyme, carbohydrate-active enzyme; ^c^AA, auxiliary activities; ^d^LPMO, lytic polysaccharide monooxygenase

The genomes of wood-decaying fungi, such as white rot fungi (e.g., *P. chrysosporium*, *T. versicolor*, *P. carnosa*, *G. subvermispora*, and *I. lacteus*) and brown rot fungi (e.g., *T. cinnabarina*, *F.s pinicola*, and *L. sulphureus*), encode a diverse repertoire of CAZymes involved in the biological decomposition of lignocellulosic biomass. This diversity is mainly attributed to a multitude of redox enzymes that act in conjunction with other CAZymes. For instance, 94, 93, 95, 66, and 71 AAs were identified in the genomes of *T. versicolor*, *P. chrysosporium*, *P. carnosa*, *G. subvermispora*, and *I. lacteus*, respectively (Table 1). Notably, the AAs/CAZymes ratios for all five white rot fungi ranged from 15.71 to 21.44%, significantly higher than those of the other 20 species evaluated in this study, except for *T. cinnabarina* (AAs/CAZymes = 17.05%). This result likely indicates robust redox activities in white rot fungi.

*P. chrysosporium* is considered the model microorganism for white rot fungi (Konan et al. 2024). Its capacity to provide economic and environmental benefits in biodegradation is largely attributed to its enzymatic arsenal, which comprises a collection of 462 CAZymes, including 192 GHs, 74 GTs, 10 PLs, 21 CEs, 72 carbohydrate-binding modules, and 93 AAs (https://www.cazy.org/e7162.html) (Drula et al. 2022). Notably, 18 out of the 93 AA genes were annotated as LPMOs, including 16 AA9 and two AA14 members (Table 1 and S2).

**Fig. 1:**
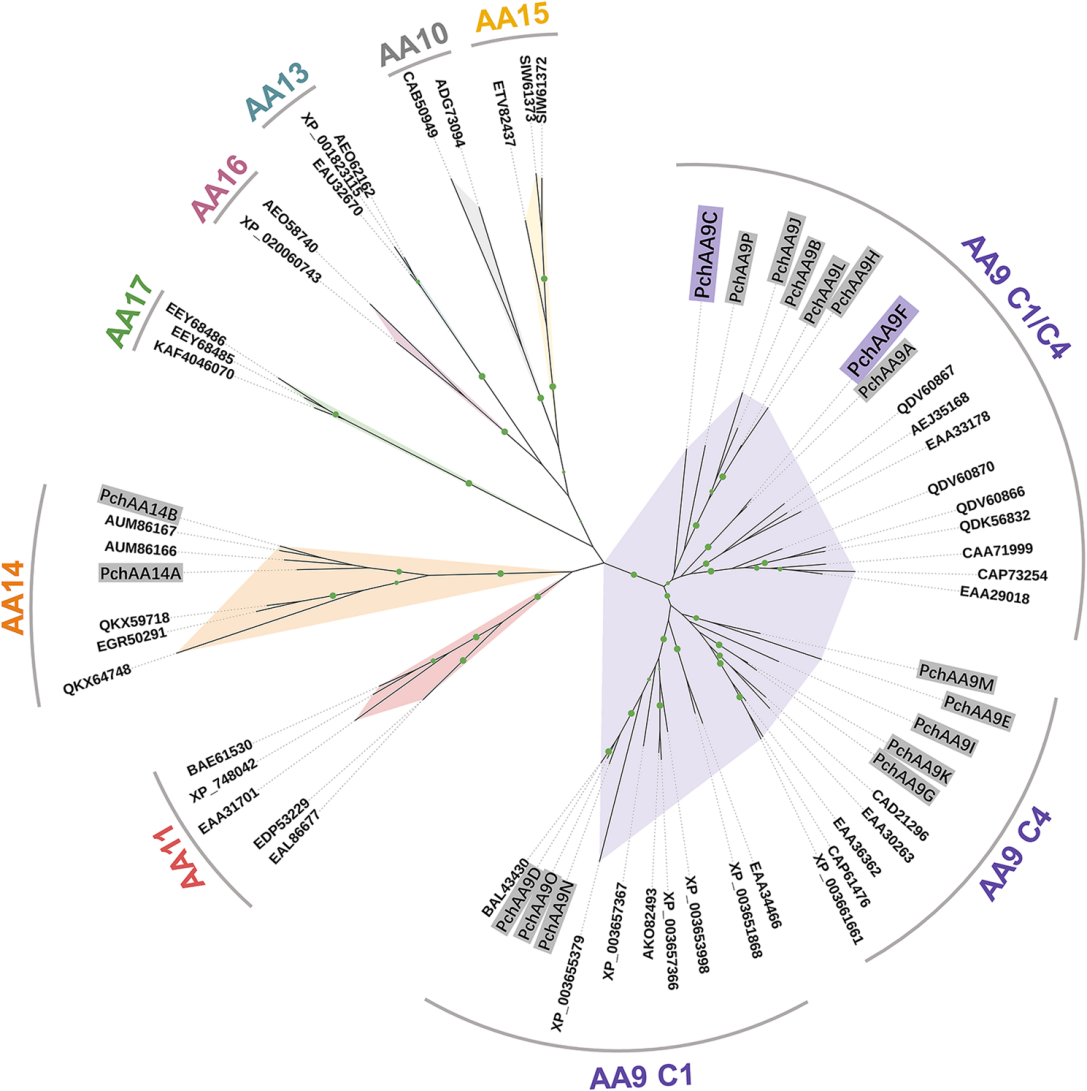
Phylogenetic tree of eighteen PchAAs and other LPMOs deposited in online databases. Amino acid sequences of the 18 PchAAs were downloaded from the Joint Genome Institute (JGI) database (https://mycocosm.jgi.doe.gov/cgi-bin/dispGeneModel?db=Phchr2&id =) and the corresponding protein IDs were listed in Table S2. Other LPMOs displayed in the phylogenetic tree are represented by their GenBank numbers. PchAA9C and PchAA9F are highlighted in violet. Green circles indicate nodes with bootstrap values greater than 80

Phylogeny analysis of the 18 PchAAs along with other characterized LPMOs revealed that 16 PchAAs clustered with characterized AA9 LPMOs (Fig. 1). Notably, the 16 PchAAs were further grouped into three sub-branches associated with C1-, C4-, and C1/C4-oxidizing activities, likely reflecting the corresponding action modes of the respective enzymes. For example, PchAA9D, PchAA9N, PchAA9O along with TtLPMO9A (GenBank: XP_003653998.1) and TtLPMO9E (GenBank: XP_003657366.1) from *T. terrestris* were clustered within the clade of C1-acting enzymes (Tõlgo et al. 2022). PchAA9E, PchAA9G, PchAA9I, PchAA9K, and PchAA9M, along with NcLPMO9A (GenBank: EAA30261.1), NcLPMO9C (GenBank: EAA36362.1), and NcLPMO9D (GenBank: CAD21296.1) from *N. crassa* were clustered within the clade of C4-acting enzymes (Isaksen et al. 2014). Among these, PchAA9D (formerly known as PcGH61D or AA9D) and PchAA9E (formerly known as PchAA9, JGI protein ID Phchr2 2,934,397) are the only functionally characterized C1- and C4-acting LPMOs, respectively, from *P. chrysosporium*, both demonstrating robust activity on cellulose (Westereng et al. 2011; Wu et al. 2013; Uchiyama et al. 2022; Frandsen et al. 2021). PchAA9A, PchAA9B, PchAA9C, PchAA9F, PchAA9H, PchAA9J, PchAA9L, and PchAA9P along with McLPMO9A (GenBank: QDV60866.1) and McLPMO9B (GenBank: QDV60867.1) from *Malbranchea cinnamomea* were grouped within the clade of C1/C4-acting enzymes (Hüttner et al. 2019). In addition, PchAA14A and PchAA14B were closely related to the xylan-oxidizing PcAA14A and PcAA14B from the white rot fungus *Pycnoporus coccineus* (Couturier et al. 2018). To discover novel LPMOs with desired activities and properties, seven *P. chrysosporium*-derived AA9 LPMOs, *PchAA9A*, *PchAA9B*, *PchAA9C*, *PchAA9F*, *PchAA9H*, *PchAA9I*, and *PcLPMO9L*, were cloned and transformed into the genome of *P. pastoris*. Among these enzymes, only PchAA9C and PchAA9F were successfully expressed in *P. pastoris*, with secreted concentrations of 10.4 and 20.7 mg/L in the culture supernatant, respectively. These yields are comparable to those reported for other fungal LPMOs produced in this expression host (O’Dell et al. 2017; Frandsen et al. 2021). Therefore, the PchAA9C and PchAA9F, which potentially possess C1/C4-oxidizing activities, were biochemically characterized in the following study.

### PchAA9C and PchAA9F are mesophilic enzymes that are activated by ascorbic acid or H_2_O_2_

The open reading frames (ORFs) of *PchAA9C* and *PchAA9F* were 987 and 939 bp, encoding proteins of 329 and 313 amino acids (AAs), respectively. SignalP 6.0 prediction (https://services.healthtech.dtu.dk/services/SignalP-6.0/) revealed that PchAA9C and PchAA9F contained signal peptides of 23 AAs and 20 AAs, respectively. HMMER analysis (https://www.ebi.ac.uk/Tools/hmmer/search/phmmer) showed that the mature peptide of PchAA9C consisted of an AA9 domain (24–244 AAs) and a CBM1 domain (293–329 AAs). Similarly, the mature peptide of PchAA9F consisted of an AA9 domain (21–245 AAs) and a CBM1 domain (277–313 AAs). Both PchAA9C and PchAA9F displayed the characteristic immunoglobulin-like fold commonly observed in AA9 LPMOs, comprising a central β-sandwich core structure with short α-helical segments positioned at the periphery (Couturier et al. 2018; Mazurkewich et al. 2021). The catalytic center of these enzymes contained a highly conserved histidine brace that served to coordinate the essential copper ion. Specifically, in PchAA9C, the copper ion was coordinated by His1, Tyr167, and His78, while in PchAA9F, the coordinating residues were His1, Tyr167, and His80 (Fig. S1).

**Fig. 2:**
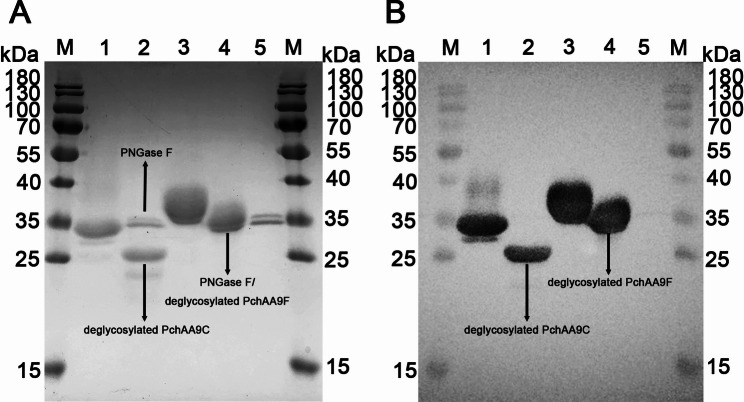
SDS-PAGE (**A**) and Western blotting (**B**) of affinity-purified native and deglycosylated PchAA9C and PchAA9F. M: marker; lane 1: native PchAA9C; lane 2: deglycosylated PchAA9C; lane 3: native PchAA9F; lane 4: deglycosylated PchAA9F; lane 5: PNGase F

Nucleotides encoding for the AA9 domains of PchAA9C and PchAA9F were introduced into the GS115 strain and induced with methanol for protein expression. After affinity-purification using 6 × His tagged Ni–NTA Sepharose, the recombinant PchAA9C and PchAA9F showed distinct bands of approximately 30 and 32 kDa, respectively (Fig. 2). It is well established that post-translational modifications (PTMs), such as glycosylation, acetylation, phosphorylation, and alkylation, play vital roles in protein folding and enzymatic catalysis in eukaryotic cells. Among these, *N*-glycosylation often contributes an additional 1–3 kDa per site to the molecular weight of target proteins (Wang et al. 2015). To determine whether the recombinant PchAA9C and PchAA9F were *N*-glycosylated, they were treated with PNGase F. Following deglycosylation, the molecular masses of PchAA9C and PchAA9F decreased by approximately 5 and 2 kDa, respectively (Fig. 2). These findings are consistent with the presence of two potential N-glycosylation sites (N59 and N233) in PchAA9C and one site (N147) in PchAA9F (https://services.healthtech.dtu.dk/services/NetNGlyc-1.0/).

**Fig. 3:**
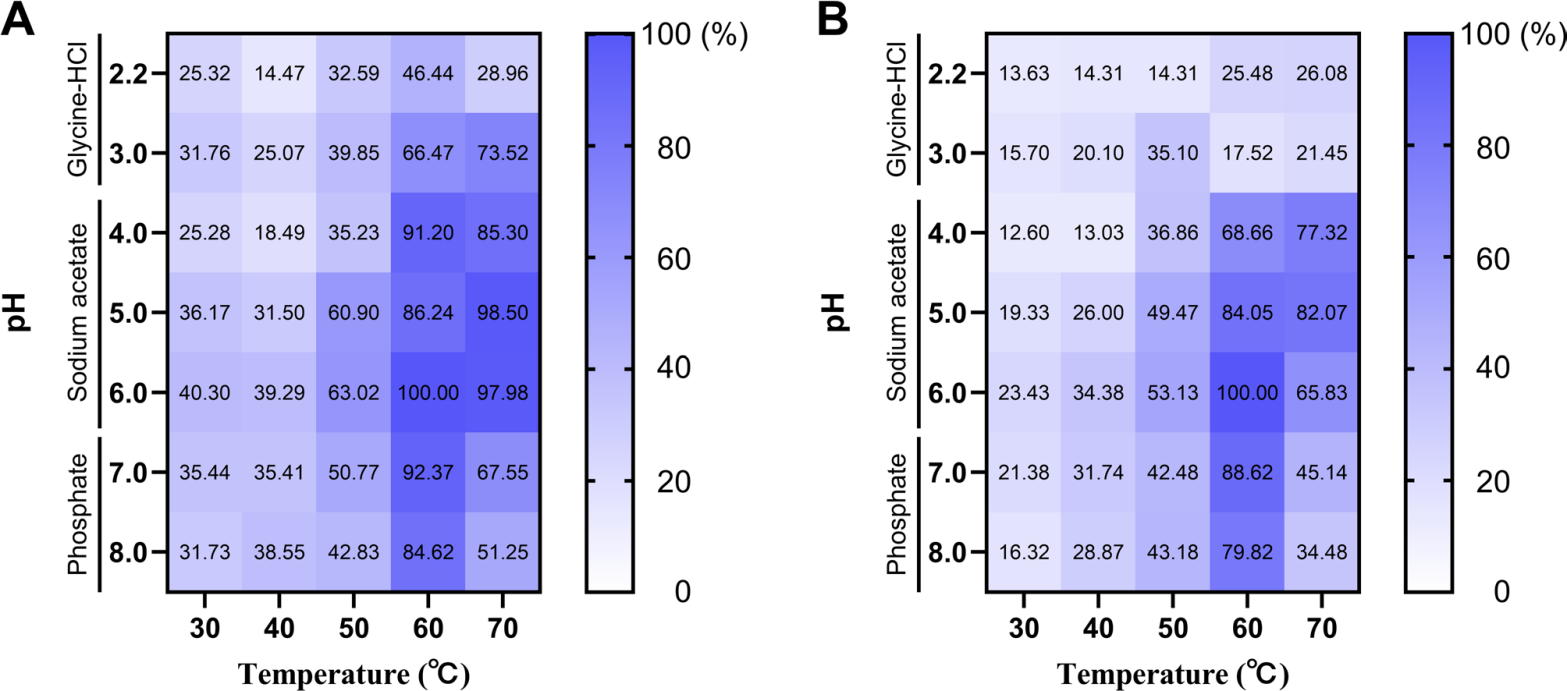
Heat map illustrating combined effects of temperature and pH on the activities of PchAA9C (**A**) and PchAA9F (**B**). The data represent the mean of three replicates (n = 3). The highest activities under the optimum conditions were established as 100%

Given the critical role of copper in oxidative activities (Fig. S2; Eijsink et al. 2019; Dan et al. 2023), both PchAA9C and PchAA9F were subjected for copper saturation. ICP-MS analysis of copper-saturated PchAA9C and PchAA9F revealed copper/PchAA9C and copper/PchAA9F ratios of 1.00 and 1.04, respectively, indicating saturation of the active sites (Sabbadin et al. 2018; Stepnov et al. 2021). PchAA9C and PchAA9F exhibited optimal temperatures of 60 °C and pH values of 6.0, respectively (Fig. 3). The PchAA9C showed relatively high activity, ranging from 50 to 70 °C (> 60%) and pH 4.0–8.0 (> 80%) (Fig. 3A). Similarly, the PchAA9F was considerably robust ranging from 60 to 70 °C (> 60%) and pH 5.0–7.0 (> 80%) (Fig. 3B). These findings suggest that PchAA9C and PchAA9F are weakly acidic, mesophilic enzymes. Most LPMOs reported in the literature exhibit optimal temperatures of 35–60 °C (Li et al. 2022; Sun et al. 2023; Raheja et al. 2024; Xu et al. 2024). However, an exception is AoAA17, an *Aspergillus oryzae* AA17 LPMO, which exhibits an optimal temperature of 100 °C (Bhatia and Yadav 2021).

**Fig. 4:**
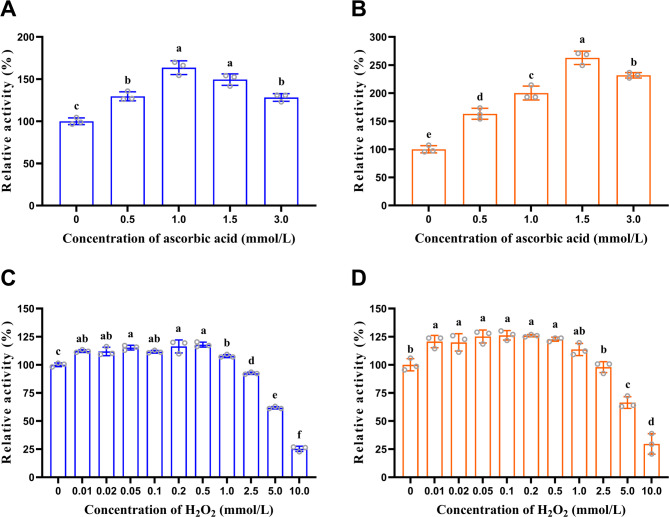
Effects of ascorbic acid and H_2_O_2_ on PchAA9C and PchAA9F activities. Effects of ascorbic acid on the activities of PchAA9C (**A**) and PchAA9F (**B**); effects of H_2_O_2_ on activities of PchAA9C (**C**) and PchAA9F (**D**). The data represent the mean ± SD (n = 3). The initial activities without ascorbic acid or H_2_O_2_ treatment were established as 100%. Statistical analysis was conducted via one-way ANOVA (Tukey’s multiple comparison test) using GraphPad Prism v.8.0. Different lowercase letters indicate the significant difference (*P* < 0.05)

The effects of ascorbic acid and H_2_O_2_ on the activities of PchAA9C and PchAA9F were evaluated (Fig. 4). The results showed that 0.5–3.0 mmol/L ascorbic acid significantly enhanced the activities of PchAA9C by 28.27–63.57% (*P* < 0.05) (Fig. 4A) and PchAA9F by 63.19–162.88% (*P* < 0.01; Fig. 4B). External reducing agents, including ascorbic acid (Sun et al. 2023; Tamburrini et al. 2024) gallic acid (Ma et al. 2024), pyrogallol (Zhang et al. 2021), sinapic acid (Chorozian et al. 2024), photocatalytic systems (Cannella et al. 2016), and enzymatic systems like cellobiose dehydrogenase (Tamburrini et al. 2024), are widely used as electron donors in LPMO-mediated oxidation reactions. In contrast, some reducing agents such as tannic acid (Frommhagen et al. 2016) and 1,2-dihydroxybenzene (Zhang et al. 2021) did not enhance LPMO catalysis. While both H_2_O_2_ and O_2_ were previously believed to serve as co-substrates for LPMO oxidation (Hangasky et al. 2018), recent studies have demonstrated that H_2_O_2_, rather than O_2_, drives LPMO to initiate the degradation of polysaccharide-like substrates (Wang et al. 2019).In this study, exposure to 0.01–0.5 mmol/L H_2_O_2_ significantly enhanced the enzymatic activities of PchAA9C by 11.57–17.90% (*P* < 0.05; Fig. 4C) and PchAA9F by 19.93–26.22% (*P* < 0.05; Fig. 4D), demonstrating superior H₂O₂ tolerance compared to ReLPMO1 and ReLPMO2 from *Rasamsonia emersonii* (Raheja et al. 2024). However, when the concentration of H_2_O_2_ exceeded 5 mmol/L, inhibitory effects on PchAA9C and PchAA9F activities were observed, likely due to oxidative damage to the LPMO enzyme (Sun et al. 2023; Kuusk et al. 2018; Calderaro et al. 2020). Additionally, CBMs in CAZymes, especially GHs and LPMOs (Cheng et al. 2020), have been shown to bind to substrates such as chitin (Liu et al. 2023), PASC (Støpamo et al. 2024), starch (Zhang et al. 2023), mannan, and xyloglucan (Liu et al. 2024). Notably, a recent study reported that MtLPMO9G-CBM, a chimera generated by fusing a CBM1 domain of MtLPMO9G to the enzyme itself, exhibited significantly improved enzymatic activity, substrate binding affinity to PASC, and tolerance to H_2_O_2_ (Gao et al. 2024).

### PchAA9C acts C1-oxidizing and PchAA9F acts as both C1- and C4-oxidizing enzyme in the breakdown of β-(Glc1 → 4Glc)-linkages

Substrate specificity analysis revealed that PchAA9C and PchAA9F were active against IML, PASC, and MCC (Table 2). Both enzymes exhibited their highest activities against IML, with specific activities of 22.1 ± 3.4 U/mg for PchAA9C and 31.7 ± 2.6 U/mg for PchAA9F. In previous studies, activities rates ranging from 0.42–14.96 U/mg where found for other AA9 LPMOs active on hemicellulose-related substrates such as carboxy-methylcellulose (CMC), IML, mixed-linkage beta-glucans, Avicel and celloligosaccharides (Frandsen et al. 2021; Agrawal et al. 2020a; Sharma et al. 2024). Surprisingly, the PMO_08942 (142.20 U/mg) and PMO_07920 (40.47 U/mg) from the thermotolerant fungus *Aspergillus terreus* 9DR displayed exceptionally high activity towards CMC (Agrawal et al. 2020b). However, neither PchAA9C nor PchAA9F showed activity against beechwood xylan, citrus pectin, citrus polygalacturonic acid colloidal chitin, konjac glucomannan, *Laminaria digitata* laminarin, or locust bean gum (data not shown). Given the substrate preferences of PchAA9C and PchAA9F, IML was selected for HPAEC-PAD and MALDI-TOF-MS analyses (Figs. S3 and 5). The results confirmed that PchAA9C and PchAA9F could release both neutral and oxidized oligosaccharides from IML. Previous studies have shown that neutral oligosaccharides, C1-, C4-, and C1/C4-oligosaccharides can be detected at retention times of 10–18 min, 17–28 min, 26–34 min, and 35–40 min, respectively, using HAPEC coupled with a CarboPac PA200 (3 × 250 mm) column (Sun et al. 2023; Tõlgo et al. 2022; Kommedal et al. 2023) or a CarboPac PA1 (3 × 250 mm) column (Corrêa et al. 2019; Forsberg et al. 2014; Kojima et al. 2016; Grieco et al. 2020) under similar elution conditions.

**Table 2:** Specific activities of PchAA9C and PchAA9F

Substrates (5 mg/mL)	Typical structures^a^	PchAA9C (U/mg protein)^b^	PchAA9F (U/mg protein)^c^
Icelandic moss lichenan		22.1 ± 3.4 (100%)^3^	31.7 ± 2.6 (100%)^3^
Barley β-glucan		6.5 ± 3.1 (29.4%)^3^	12.8 ± 2.9 (40.4%)^3^
Phosphoric acid swollen cellulose		14.3 ± 2.5 (64.7%)^3^	13.3 ± 1.2 (42.0%)^3^
Microcrystalline cellulose		4.3 ± 0.6 (19.5%)^3^	3.3 ± 0.7 (10.4%)^3^

^a^The blue circles indicate glucose units

^b^Data represent the mean ± SD (n = 3)

^c^The highest activity against the Icelandic moss lichenan was established as 100%

**Fig. 5:**
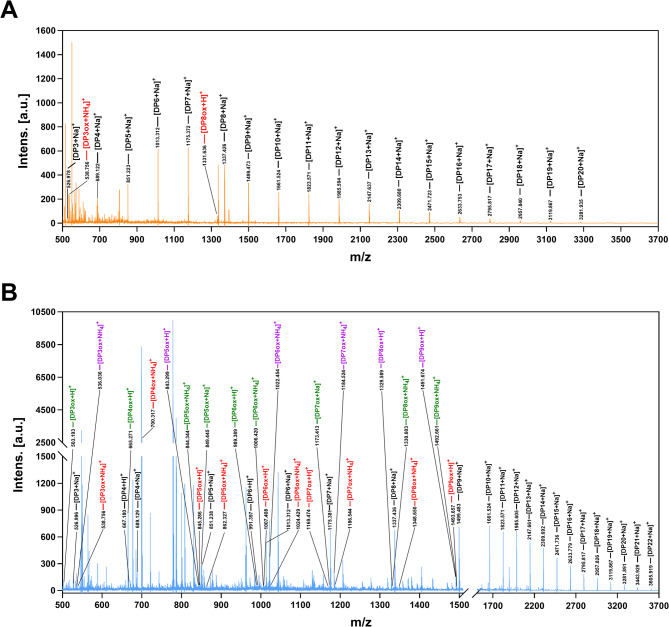
MALDI-TOF-MS spectra of products released from Icelandic moss lichenan by PchAA9C (**A**) and PchAA9F (**B**). Peaks of neutral, C1-oxidized, C4-oxidized, and C1/C4 double-oxidized products were labeled with black, red, green and purple, respectively. The peaks [M + H]⁺, [M + Na]⁺, and [M + NH_4_]⁺ correspond to the protonated molecular ion, the sodium adduct, and the ammonium adduct molecular ions, respectively

To further understand the cleavage profiles and catalytic products of PchAA9C and PchAA9F, the molecular masses of the oligosaccharides were determined using MALDI-TOF-MS. As shown in Fig. 5A, PchAA9C released a series of neutral cello-oligosaccharides with a degree of polymerization (DP) ranging from 3 to 20 from IML. In contrast, only two oxidized cello-oligosaccharides were detected, with m/z signals at 538.8 (values of [M + NH_4_ Da]^+^) and 1331.6 (values of [M + H Da]^+^) corresponding to the C1-oxidized cellotriose (DP3ox) and DP8ox with a terminal C1-specific aldonic acid (Zhang et al. 2021). Even though PchAA9C was grouped within the clade of C1/C4-oxidizing LPMOs (Fig. 1), the MALDI-TOF-MS results (Fig. 5A) indicated that PchAA9C exclusively cleaved the C1 position of sugars within the main chain of the substrate. This acting mode resembles that of PchAA9D (previously referred to as PcGH61D) (Westereng et al. 2011; Wu et al. 2013).

Interestingly, compared to PchAA9C, PchAA9F released a broader spectrum of neutral cello-oligosaccharides with DPs of 3–22 from IML, along with seven oxidized cello-oligosaccharides. These oxidized products had m/z signals at 538.8 ([M + NH_4_ Da]^+^), 700.3 ([M + NH_4_ Da]^+^), 845.3 ([M + H Da]^+^)/862.3 ([M + NH_4_ Da]^+^), 1007.4 ([M + H Da]^+^)/1024.4 ([M + NH_4_ Da]^+^), 1169.5 ([M + H Da]^+^)/1186.5 ([M + NH_4_ Da]^+^), 1348.7 ([M + NH_4_ Da]^+^), and 1493.7 ([M + H Da]^+^) corresponding to the C1-oxidized DP3ox–DP9ox (Fig. 5B) (Kojima et al. 2016; Corrêa et al. 2019). PchAA9F generated seven C4-oxidized products with the following m/z signals: 503.2 ([M + H Da]^+^), 665.3 ([M + H Da]^+^), 844.3 ([M + NH_4_ Da]^+^)/849.4 ([M + Na Da]^+^), 989.4 ([M + H Da]^+^)/1006.4 ([M + NH_4_ Da]^+^), 1173.4 ([M + Na Da]^+^), 1330.6 ([M + NH_4_ Da]^+^), and 1492.7 ([M + NH_4_ Da]^+^) corresponding to the C4-oxidized DP3ox–DP9ox. In addition, six double-oxidized cello-oligosaccharides were detected in the products of PchAA9F catalysis, with m/z signals of 536.0 ([M + NH_4_ Da]^+^), 843.3 ([M + H Da]^+^), 1022.5 ([M + NH_4_ Da]^+^), 1184.5 ([M + NH_4_ Da]^+^), 1329.6 ([M + H Da] +), and 1491.7 ([M + H Da] +) corresponding to the C1-/C4-double-oxidized DP3ox, DP5ox, DP6ox, DP7ox, DP8ox, and DP9ox, respectively (Fig. 5B) (Forsberg et al. 2014; Grieco et al. 2020). Therefore, PchAA9F oxidized both C1 and C4 positions of sugars within the main chains of β-1, 4-linked substrates. Compared to LPMOs that exclusively oxidize at C1 or C4 positions, C1/C4 double-oxidizing LPMOs, such as PchAA9F, are of greater interest for industrial applications due to their ability to introduce multiple oxidation points on substrates.

### PchAA9C and PchAA9F enhance the saccharification effects of a GH cocktail on natural lignocellulosic materials

**Fig. 6:**
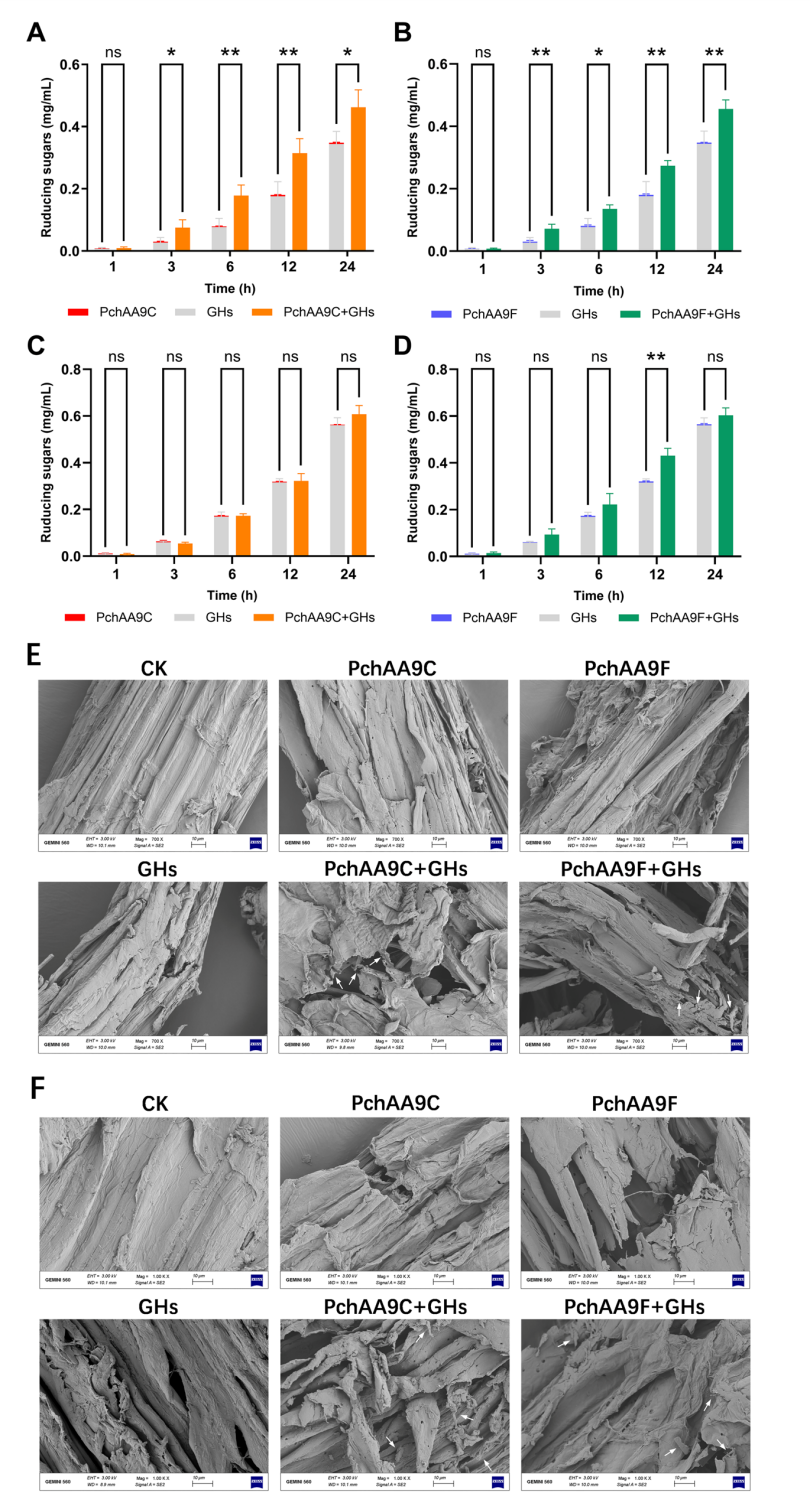
Enzymatic degradation of natural lignocellulosic feedstocks. Time-course of reducing sugars released from corn straw by PchAA9C (**A**) and PchAA9F (**B**). Time-course of reducing sugars released from soybean straw by PchAA9C (**C**) and PchAA9F (**D**). **E** SEM images of untreated and enzyme-treated corn straw; **F** SEM images of untreated and enzyme-treated soybean straw. Statistical significance was conducted using Student’s *t*-test. *, *P* < 0.05; **, *P* < 0.01

To investigate the synergistic effects of PchAA9C and PchAA9F in cooperation with GHs on substrate degradation, the recombinant enzymes were tested in combination with a GH cocktail. This cocktail included the bifunctional endo-/exo-xylanase XYN-LXY (Wang et al. 2015), the bifunctional endo-/exo-glucanase Cel5A-h38 (Cao et al. 2021), and the exo-polygalacturonase IDSPGA28-16 (Deng et al. 2023) for the deconstruction of *Z. mays* L. and *G. max* L. straws (Fig. 6). The total reducing sugars released by the GH cocktail alone and in combination with PchAA9C or PchAA9F were compared to assess their synergistic effects. The GH cocktail alone released reducing sugars from *Z. mays* L. straw at the early stage of reaction (1–3 h; Fig. 6A and B). Sugar concentrations increased over time, reaching 0.34 ± 0.04 mg/mL after 24 h. However, the addition of PchAA9C or PchAA9F significantly enhanced saccharification, increasing sugar production by 1.34-fold (*P* < 0.05) and 1.33-fold (*P* < 0.01), respectively, after 24 h. This result demonstrates the synergy between LPMO and GHs in the depolymerization of *Z. mays* L. straw. A recent study demonstrated that supplementing a cellulase cocktail with two C1/C4-oxidizing LPMOs from *R. emersonii* (ReLPMO1 and ReLPMO2) increased reducing sugar yields from rice straw by 19.01% and 15.66%, respectively (Raheja et al. 2024). In contrast, the C1/C4-oxidizing PchAA9F in this study exhibited a more substantial enhancement in saccharification efficiency (Fig. 6B). For *G. max* L. straw, the GH cocktail alone effectively released sugars. However, the addition of PchAA9C (*P* = 0.06) or PchAA9F (*P* = 0.09) did not result in a statistically significant improvement in the production of reducing sugars after 24 h (Fig. 6C and D). This result could be attributed to the differences in spatial structure and chemical composition of the lignocellulosic materials. To better understand these effects, the morphologies of *Z. mays* L. and *G. max* L. straws were investigated using SEM. The results showed that native *Z. mays* L. and *G. max* L. straws had relatively smooth surfaces and intact cell walls. After treatment with the GH cocktail, the fiber structures were mildly disrupted. Notably, the addition of PchAA9C and PchAA9F caused more severe degradation (indicated by arrows in Fig. 6E and F). In conclusion, the addition of PchAA9C and PchAA9F resulted in a modest enhancement of the saccharification efficiency of the GH cocktail on plant-derived feedstocks.

Due to their versatile effects on polysaccharide depolymerization, LPMOs are widely used in the degradation and biorefinery of lignocellulosic materials (Calderaro et al. 2020; Raheja et al. 2024). The ability of LPMOs to enhance the saccharification of natural lignocellulosic materials, such as agricultural waste from rice (Sun et al. 2023; Raheja et al. 2024), wheat (Long et al. 2021), maize (Park et al. 2023), and peanut (Deng et al. 2024), which are composed of cellulose, hemicellulose, and pectin in their cell walls, has been extensively validated. In this study, both PchAA9C and PchAA9F enhanced substrate degradation by a GH cocktail containing glucanase, xylanase, and pectinase (Fig. 6), in agreement with previously reported findings. Notably, in comparison to the exclusive C1-acting PchAA9C (Fig. 5A), PchAA9F, which acts as a C1- and C4-oxidizing enzyme (Fig. 5B), was more effective in producing reducing sugars from soybean straw during the early stages of reaction, particularly between 6 and 12 h (Fig. 6B). The observed differences in substrate depolymerization performance suggests that the dual oxidation activity of PchAA9F at both C1 and C4 positions likely resulted in more pronounced synergistic effects with GHs.

**Fig. 7:**
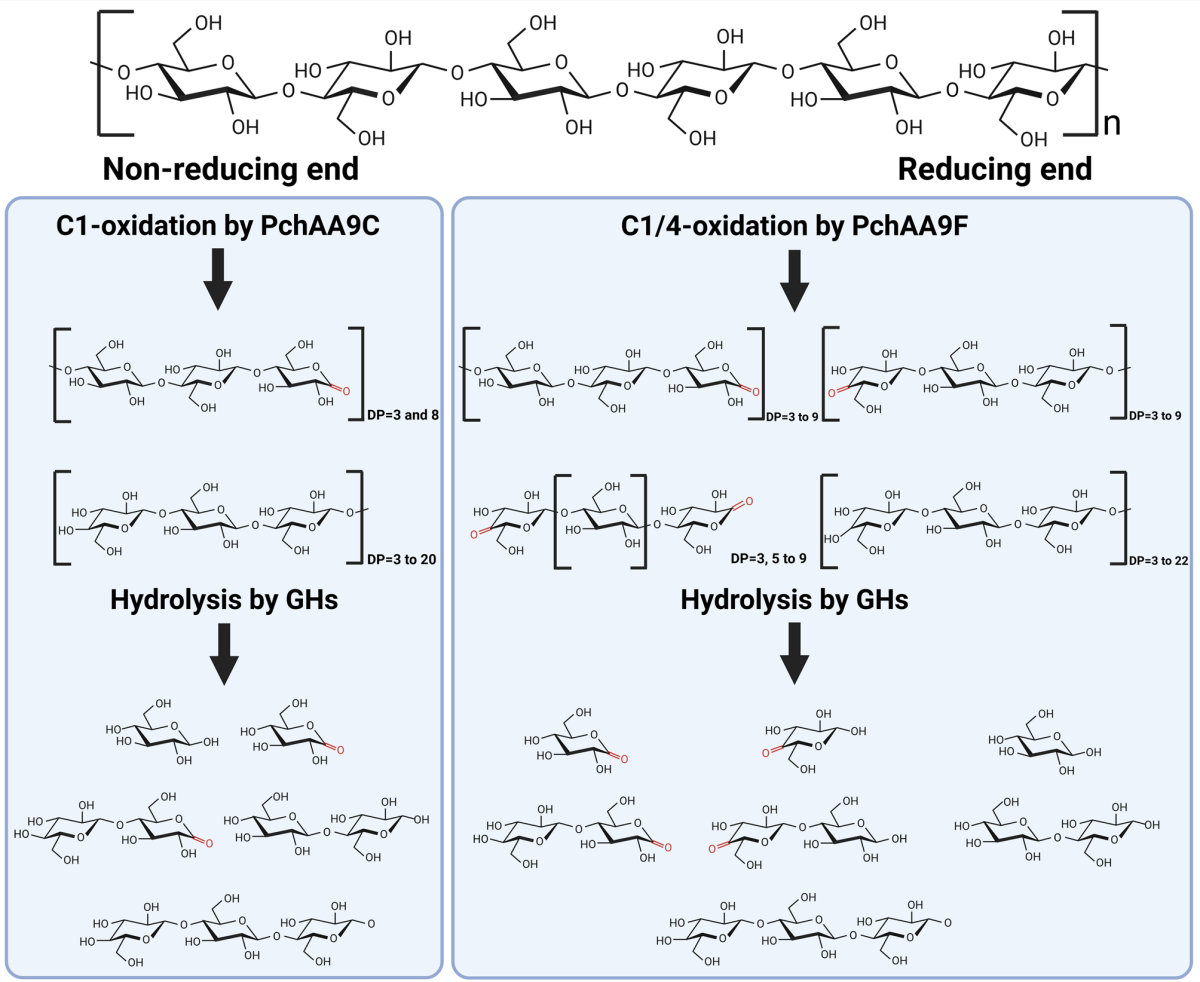
Proposed synergistic mechanism between PchAA9C/PchAA9F and glycoside hydrolase cocktails in substrate degradation

Contrary to the general understanding of synergistic effects between LPMOs and GHs, some LPMOs and GHs did not exhibit such effects under specific co-reaction conditions. For example, the core cellulolytic enzymes MtCel5A (a cellulase) and MtCel7A (a cellobiohydrolase) in *Myceliophthora thermophila* (also known as *T. thermophilus*) were inhibited by the C1-oxidizing MtLPMO9E and C4-oxidizing MtLPMO9J, but were enhanced by the C1/C4-double-oxidizing MtLPMO9H (Qin et al. 2022). In another instance, a C1-oxidizing TtAA9E displayed a greater synergy with cellulases on acid-pretreated rice straw compared to a C1/C4-double-oxidizing TtAA9A (Kim et al. 2017). Conversely, TtAA9A exhibited a more pronounced synergism than TtAA9E on alkali-pretreated rice straw. These findings are consistent with the results obtained in this study, where PchAA9C (C1-oxidizing) and PchAA9F (C1/C4-double-oxidizing) showed varying synergistic effects depending on the substrate (Fig. 6B). The above findings support a proposed mechanism underlying the synergistic action of PchAA9C/PchAA9F with the GH cocktail used in this study (Fig. 7). However, the detailed mechanisms underlying these variations remain to be further elucidated.

## Conclusions

This study focused on the identification and biochemically characterization of two novel AA9 LPMOs, PchAA9C and PchAA9F, from the model white rot fungus *P. chrysosporium*. PchAA9C and PchAA9F are mesophilic enzymes active towards substrates composed of β-(Glc1 → 4Glc)-linkages. Compared to the exclusive C1-oxdizing PchAA9C, the C1/C4-double-oxidizing PchAA9F demonstrated more pronounced effects in enhancing the saccharification effects of a GH cocktail on natural agricultural wastes. To our knowledge, PchAA9F is the first C1/C4-double-oxidizing LPMO isolated from *P. chrysosporium*, making it particularly promising for applications in biorefinery and bioconversion of plant-derived biomass.

## Supplementary Information

Below is the link to the electronic supplementary material.

Supplementary Material 1

## Data Availability

All data generated or analyzed during this study are included in this published article.

## Abbreviations

LPMOs: Lytic polysaccharide monooxygenases
AA: Auxiliary activity
GHs: Glycoside hydrolases
PLs: Polysaccharide lyases
CEs: Carbohydrate esterases
CBMs: Catalytic carbohydrate-binding modules
CAZymes: Carbohydrate-active enzymes
IML: Icelandic moss lichenan
PASC: Phosphoric acid swollen cellulose
HPAEC-PAD: High performance anion-exchange chromatography with a pulsed
