# Supplementary material for: Identification and characterization of two AA9 lytic polysaccharide monooxygenases isolated from the enzymatic arsenal in Phanerochaete chrysosporium
Source: Bioresour Bioprocess. 2025 Oct 10;12(1):112. doi: 10.1186/s40643-025-00950-0 (PMC12514121; doi:10.1186/s40643-025-00950-0)
Supplement: Supplementary file 1 — Supplementary Material 1 [file 40643_2025_950_MOESM1_ESM.docx]

**Supplementary materials**

**
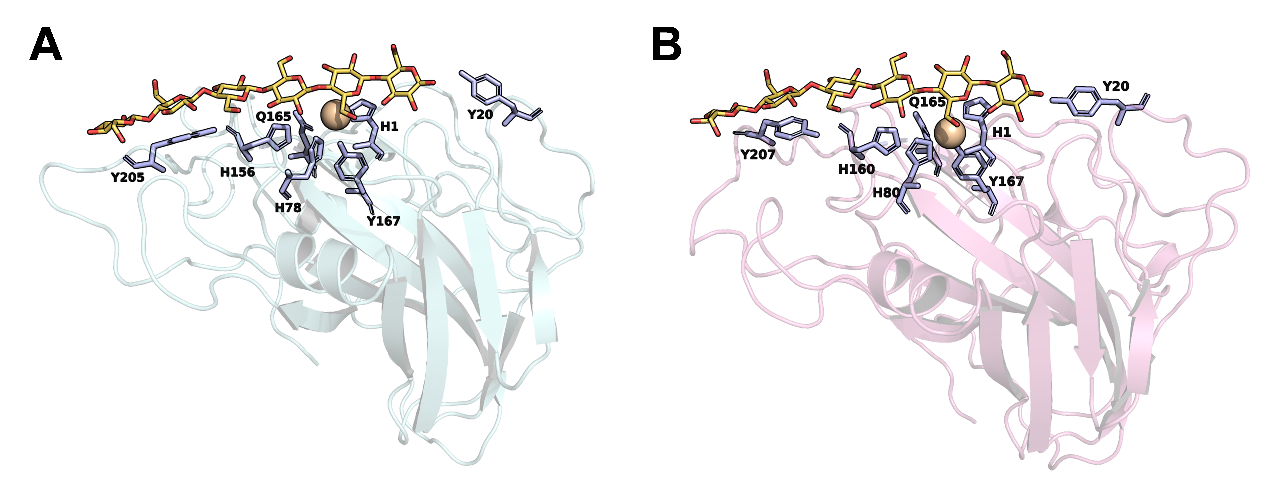
**

**Figure S1.** Three dimensional structures of PchAA9C (A) and PchAA9F (B) showing conserved substrate-binding grove with modelled cellohexaose. Copper ion were shown as a brown sphere. Key residues potentially involved in substrate binding were displayed as sticks.

**
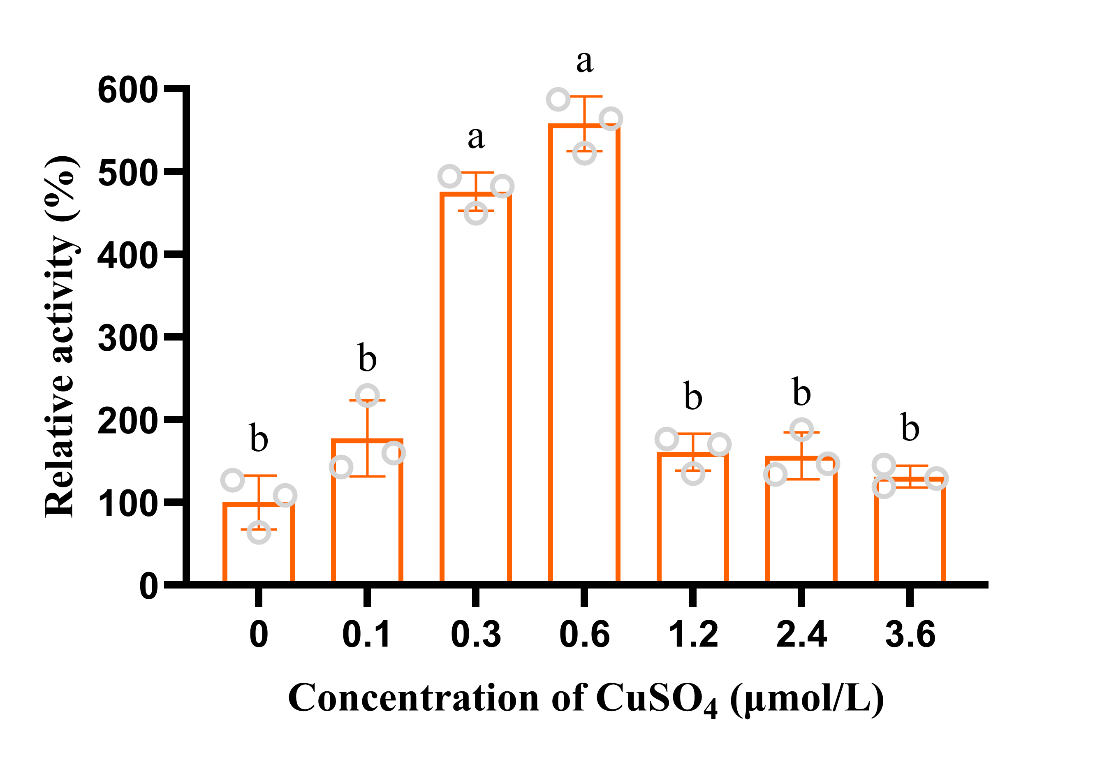
**

**Figure S2.** Effects of copper concentration on the activity of PchAA9F. The data represent the mean ± SD (n = 3). The initial activity without copper treatment was established as 100%. Statistical analysis was conducted via one-way ANOVA (Tukey’s multiple comparison test) using GraphPad Prism v.8.0. Different lowercase letters indicate the significant difference (*P* < 0.05).

**
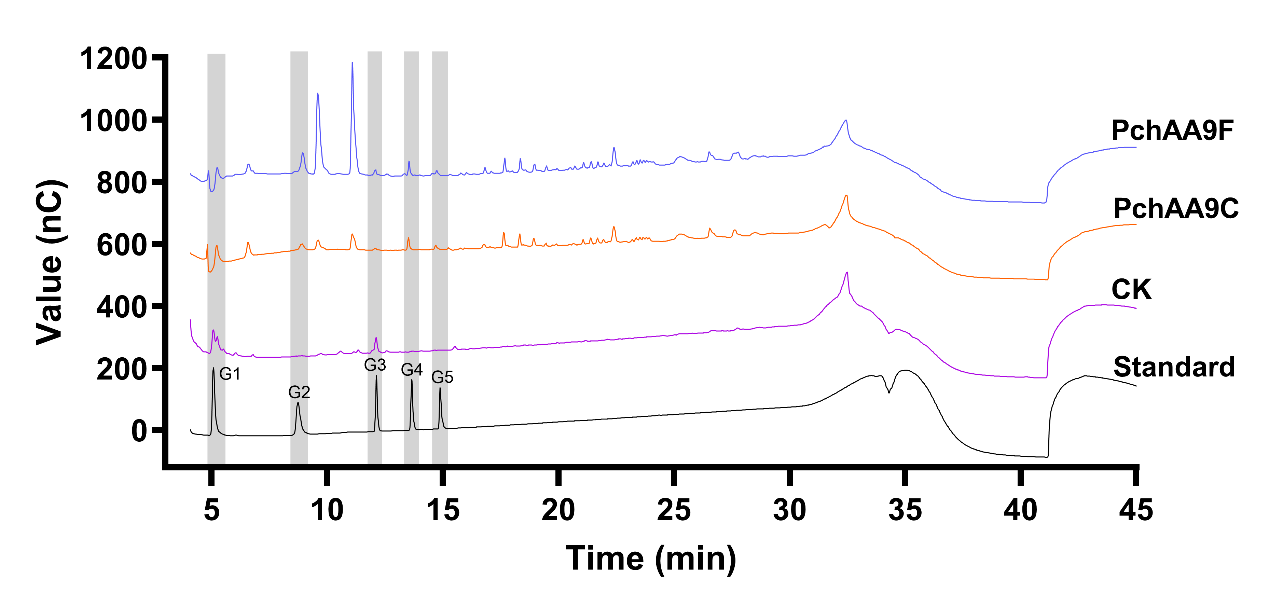
**

**Figure S3.** HPAEC chromatograms of the degradation products from Icelandic moss lichenan by PchAA9C or PchAA9F. G1, glucose; G2, cellobiose; G3, cellotriose; G4, cellotetraose; G5, cellopentaose.

**Table S1.** Oligonucleotides used in this study

| **Primers** | **Sequence (5’ to 3’)^1^** | **Length (bp)** | **Purposes** |
| --- | --- | --- | --- |
| PchAA9C -F | CGTAGAATTCCCTAGGGTTATCACGAACTACGCGACGTAC | 705 | Amplification of *PchAA9C* |
| PchAA9C -R | CTAAGGCGAATTAATTCGCGGCCGCGTGATGATGATGATGATGAAAGACGTCCTGGGCCCA |  |  |
| PchAA9F-F | GGCTGAAGCTTACGTAGAATTCCACGGCGGTGTCCTCTC | 735 | Amplification of *PchAA9F* |
| PchAA9F-R | CTAAGGCGAATTAATTCGCGGCCGCGTGATGATGATGATGATGAGAGGTGGGACCAACGGA |  |  |

Restriction sites were underlined, and the sequence of 6 × His tags were shaded.

**Table S2** Eighteen lytic polysaccharide monooxygenases (LPMOs) annotated in the genome of *Phanerochaete chrysosporium*^1^

| **Protein names** | **Protein Ids** | **Families** | **References** |
| --- | --- | --- | --- |
| PchAA9A | Phchr2 2976448 | Auxiliary activity family 9 | NA |
| PchAA9B | Phchr2 2982894 | Auxiliary activity family 9 | NA |
| PchAA9C | Phchr2 3003776 | Auxiliary activity family 9 | This study |
| PchAA9D | Phchr2 2908293 | Auxiliary activity family 9 | Westereng et al., 2011  Wu et al., 2013  Uchiyama et al., 2022 |
| PchAA9E | Phchr2 2934397 | Auxiliary activity family 9 | Frandsen et al., 2021 |
| PchAA9F | Phchr2 2980158 | Auxiliary activity family 9 | This study |
| PchAA9G | Phchr2 3005492 | Auxiliary activity family 9 | NA |
| PchAA9H | Phchr2 1216344 | Auxiliary activity family 9 | NA |
| PchAA9I | Phchr2 3027731 | Auxiliary activity family 9 | NA |
| PchAA9J | Phchr2 3040475 | Auxiliary activity family 9 | NA |
| PchAA9K | Phchr2 3029242 | Auxiliary activity family 9 | NA |
| PchAA9L | Phchr2 42616 | Auxiliary activity family 9 | NA |
| PchAA9M | Phchr2 2975821 | Auxiliary activity family 9 | NA |
| PchAA9N | Phchr2 2982319 | Auxiliary activity family 9 | NA |
| PchAA9O | Phchr2 3004691 | Auxiliary activity family 9 | NA |
| PchAA9P | Phchr2 3026777 | Auxiliary activity family 9 | NA |
| PchAA14A | Phchr2 2949697 | Auxiliary activity family 14 | NA |
| PchAA14B | Phchr2 2981594 | Auxiliary activity family 14 | NA |

^1^ The sequences deposited in the Joint Genome Institute (JGI) database (https://mycocosm.jgi.doe.gov/cgi-bin/dispGeneModel?db=Phchr2&id=) were annotated by Drula et al., (2022).

NA, not available.

Reference:

Drula E, Garron ML, Dogan S, Lombard V, Henrissat B, Terrapon N (2022) The carbohydrate-active enzyme database: functions and literature. Nucleic Acids Res 50(D1):D571−D577. https://doi.org/10.1093/nar/gkab1045

Frandsen KEH, Haon M, Grisel S, Henrissat B, Lo Leggio L, Berrin JG (2021) Identification of the molecular determinants driving the substrate specificity of fungal lytic polysaccharide monooxygenases (LPMOs). J Biol Chem 296:100086. https://doi.org/10.1074/jbc.RA120.015545

Westereng B, Ishida T, Vaaje-Kolstad G, Wu M, Eijsink VGH, Igarashi K, Samejima M, Ståhlberg J, Horn SJ, Sandgren M (2011) The putative endoglucanase PcGH61D from *Phanerochaete chrysosporium* is a metal-dependent oxidative enzyme that cleaves cellulose. PLoS One 6(11):e27807. https://doi.org/10.1371/journal.pone.0027807

Wu M, Beckham GT, Larsson AM, Ishida T, Kim S, Payne CM, Himmel ME, Crowley MF, Horn SJ, Westereng B, Igarashi K, Samejima M, Ståhlberg J, Eijsink VGH, Sandgren M (2013) Crystal structure and computational characterization of the lytic polysaccharide monooxygenase GH61D from the Basidiomycota fungus *Phanerochaete chrysosporium*. J Biol Chem 288(18):12828−12839. https://doi.org/10.1074/jbc.M113.459396

Uchiyama T, Uchihashi T, Ishida T, Nakamura A, Vermaas JV, Crowley MF, Samejima M, Beckham GT, Igarashi K (2022) Lytic polysaccharide monooxygenase increases cellobiohydrolases activity by promoting decrystallization of cellulose surface. Sci Adv 8(51):eade5155. https://doi.org/10.1126/sciadv.ade5155
